# Two sides of the same coin: the N-terminal and the receptor binding domains of SARS-CoV-2 Spike

**DOI:** 10.2217/fvl-2022-0181

**Published:** 2023-03-06

**Authors:** Massimo Ciccozzi, Stefano Pascarella

**Affiliations:** ^1^Medical Statistic & Molecular Epidemiology Unit, University of Biomedical Campus, Rome, Italy; ^2^Department of Biochemical Sciences ‘A Rossi Fanelli’, Sapienza Università di Roma, Rome, 00185, Italy

**Keywords:** allosteric interaction, AXL, electrostatic potential, indels, IntAct, N-terminal domain, receptor binding domain, SARS-CoV-2, sialoside, Spike

## Abstract

The SARS-CoV-2 Spike receptor binding domain and N-terminal domain interact with each other in an intricate mechanism. Mutations modulate the interplay between the Spike and host molecules. This editorial comments on the intricacies of SARS-CoV-2 Spike interactions.

The SARS-CoV-2 Spike protein is the main player in host selection, cell adhesion, tissue tropism and infection in COVID-19 [1]. As such, it is a fundamental viral determinant of the development of the COVID-19 pandemic. The spike protein is a glycosylated homotrimer in which each monomer is organized in different domains, each with a specific function. After interaction with extracellular and membrane components of host cells, this sophisticated molecular machine undergoes proteolytic cleavage and transitions between different conformational and functional states. These transitions enable virus entry into the cell.

The Spike receptor binding domain (RBD) has been under intense scrutiny since the onset of the pandemic because of its role in the host cell invasion process, during which it binds the angiotensin-converting enzyme 2 (ACE2) receptor on the host cell surface. This process is similar to invasion by SARS-CoV and HCoV-NL63 [2]. The RBD contains two domains: a large core domain, which folds as a twisted five-stranded antiparallel β-sheet, and the receptor binding motif (RBM), a 69-residue long insertion of two short α-helices and β-strands between the β4 and β7 strands. The RBM specifically interacts with the ACE2 receptor. Similarities between the RBD and two bacterial proteins, the nicking enzyme from *Staphylococcus aureus* and the mobilization protein A from *Pseudomonas aeruginosa* (Protein Data Bank codes 4HT4 and 2NS6, respectively), have been observed. A common evolutionary origin is unlikely considering the very low sequence similarity (∼4%) and the lack of any disulfide bridge structurally equivalent to those present in the RBD.

The RBD, particularly the RBM, is the main target of mutations that characterize SARS-CoV-2 variants, some of which have spread rapidly throughout human populations and are thus called variants of concern (VOC). Specific mutations can alter the properties of the RBD, influencing its interaction with ACE2 and contribute to the virus’s ability to evade host immune responses and vaccine induced immunity [3]. It has been suggested that during the evolution of the most widespread VOCs, RBD mutations tended to increase the positivity of the RBD’s surface electrostatic potential in comparison to the original strain of the virus that emerged in Wuhan, China. This change in surface electrostatic potential possibly modulates binding to the predominantly negative charge of the ACE2 surface [4–6] and may influence binding to other receptors and biomolecules due to the important role of electrostatic forces in biomolecular interaction and recognition. A proposal has been recently published about the role of electrostatic interactions of the RBD with ACE2 on binding affinity and VOC transmissibility - using a sophisticated theoretical model, the authors found a linear correlation between the total charge of the RBD and the predicted affinity for ACE2 and suggested that this ‘charge rule’ can be a quick and easy predictor of variant transmissibility and infectivity [7]. There is a similar correlation between net charge and the estimated effective reproduction number (R_t_) [8]. In addition, a change in surface net charge may alter interactions with the immune system and other cell molecular components such as glycans. Indeed, experimental evidence is emerging that acidic glycans serve as co-receptors for SARS-CoV-2 and that the RBD recognizes several classes of oligosaccharides containing sialic acid [9].

It is noteworthy that, so far, no insertions and/or deletions (indels) have been observed in the RBDs of the known variants. However, the evolution and adaptation of β-coronaviruses imply structural changes in the RBD through the occurrence of several indels, particularly in the RBM. Evolutionary conservation analyses carried out with the program ConSurf [10] confirm that the RBM is the least conserved portion of the RBD. The resistance of the RBD of SARS-CoV-2 variants to indels suggests that this domain is subject to stringent structural and functional constraints, such that remodeling of the peptide backbone is not tolerated and will allow only point mutations in emerging variants. This clearly has implications for pathogenesis and vaccine development.

The N-terminal domain (NTD) of the SARS-CoV-2 Spike protein has received much less attention, though it is an equally important component of this protein. It has a galectin-like fold, and several authors have suggested that the domain can bind sialoside moieties of cell surface components [11,12]. It may also bind other accessory cell receptors, such as several lectin receptors and/or the extracellular immunoglobulin domain of AXL (‘anexelekto’) receptor tyrosine kinase [13]. It is known that the Spike NTD of other coronaviruses can bind specific receptors or sugar receptors. For example, the mouse hepatitis coronavirus NTD interacts with the carcinoembryonic antigen related cell adhesion molecule 1 (CEACAM1) that contains two or four immunoglobulin domains. The NTDs of the bovine and the human OC43 coronaviruses recognize the sugar 5-*N*-acetyl-9-*O*-acetylneuraminic acid (Neu5, 9Ac2) [2]. The NTDs of the Spike protein of other coronavirus genera (α, γ and δ) are evolutionarily and structurally related to those of the β-coronaviruses and recognize, with diverse specificities, sugar receptors different from Neu5, 9Ac2 [2]. Interaction with accessory receptors may influence host selection, tissue tropism and pathogenicity. Moreover, the NTD is an important target for host immune responses and for selective gene pressure, as suggested by numerous mutations that have occurred in the NTDs of VOCs, some of which confer evasion from protective antibody responses. Indeed, an NTD electropositive supersite targeted by neutralizing antibodies has been described [14]. Interestingly, unlike the RBD, the SARS-CoV-2 NTD fold appears structurally more flexible as it is prone to accept indels within its loops and strands, in addition to point mutations. Several of these indels affect the NTD sites potentially important for molecular interactions. For example, the deletion of amino acids 69/70 occurs in the NTD's N2 loop that is predicted to interact with sialosides. This deletion has been observed in Omicron lineages BA.1, BA.3, BA.4 and BA.5. The deletions of 143/144 and 136–144 have also been reported in the BA.1 and BA.3 lineages and B.1.640.2, respectively. These deletions occur in the N3 loop, while the 246–252 deletion in the C.37 variant hits the NTD strands flanking the N5 loop, located in the putative site of interaction with AXL [15]. Point mutations can edit the glycosylation state of the domain, as in the case of the lambda variant, where the D253N mutation adds a potential N-glycosylation site [15]. It may be speculated that all these mutations fine-tune the affinity and specificity of the NTD for different molecular and extracellular components and interaction with the host immune system. A recently published work reports a comprehensive study on the structural and functional properties of the NTD loops [16]. Using a combination of experimental and theoretical analyses, the authors pointed out that modification of NTD loop length, particularly of loop N2, can alter the properties of the Spike protein and can modulate the efficacy of virus cell entry, interaction with the immune system, adaptation to a new host as well as protein stability. Accordingly, ConSurf analysis shows that the NTD loops are most variable during the evolution of β-coronaviruses.

Changes in the NTD’s surface electrostatic potential during variant evolution should be considered. It has been noticed that, overall, the NTDs of variants tend to maintain a net charge close to neutrality or moderate positivity irrespective of the presence of indels or point mutations [8,17]. However, this trend changed with the first Omicron variant BA.1, whose NTD has a negative net charge caused by the Glu-Pro-Glu insertion at sequence position 214. This insertion was lost in all successive Omicron subvariants. The most recent XBB and XBB.1 variants also display an NTD with a net negative charge caused by the mutations His146Gln and Gln183Glu. The functional implications of these alterations are not clear and should be studied further. Very likely, they have a role in host tissue tropism and may influence interaction with the RBD and the host immune system.

The overall effect of the structural changes on the RBD and NTD is considerably complicated, according to the observations of several authors of a long-range allosteric interaction between the NTD and RBD [18,19]. Indels or point mutations in the NTD could therefore influence the properties of the RBD and vice versa, the entire Spike protein and its pathogenic characteristics.

A search in the databank IntAct, which collects known protein–protein interactions, indicates that the SARS-CoV-2 Spike protein can directly interact with at least 20 proteins or small molecules besides ACE2 [20]. These interactions have been selected with a Mi score equal to 0.6. This score measures the reliability of the existence of the interaction based on the experimental or theoretical data, number of publications and type of interactions, and ranges from 0 to 1 with 1 being the best. The threshold of 0.6 should select the most likely interactions. For a comparison, lowering the Mi score threshold to 0.5 increases the number of interactors to about 70. The Spike protein can interact with a range of different biomolecules like lectins, receptors (e.g., AXL), cationic peptides such as defensins, platelet-activating receptors such as CD42b and proteases, to name a few.

The combination of mutations on the NTD and RBD of the Spike protein can have complex effects on these interactions and can confer different degrees of pathogenicity and immune evasion to the virus. Studies on the structural and functional properties of the Spike protein can shed light on the molecular mechanisms underlying the biology of SARS-CoV-2 and other similar, emerging viruses.
